# The Differential Impact of a Response’s Effectiveness and its Monetary Value on Response-Selection

**DOI:** 10.1038/s41598-020-60385-9

**Published:** 2020-02-25

**Authors:** Noam Karsh, Eitan Hemed, Orit Nafcha, Shirel Bakbani Elkayam, Ruud Custers, Baruch Eitam

**Affiliations:** 1 Department of Psychology, University of Haifa, Israel, Mount Carmel, Haifa 31905 Israel; 2 Department of Psychology, Tel-Hai Academic College, Qiryat Shemona, 1220800 Israel; 3 Department of Psychology, Utrecht University, Heidelberglaan 1, 3584 CS Utrecht, Netherlands

**Keywords:** Motivation, Motivation, Motor control, Reward, Human behaviour

## Abstract

While known reinforcers of behavior are outcomes that are valuable to the organism, recent research has demonstrated that the mere occurrence of an own-response effect can also reinforce responding. In this paper we begin investigating whether these two types of reinforcement occur via the same mechanism. To this end, we modified two different tasks, previously established to capture the influence of a response’s effectiveness on the speed of motor-responses (indexed here by participants’ reaction times). Specifically, in six experiments we manipulated both a response’s ‘pure’ effectiveness and its outcome value (e.g., substantial versus negligible monetary reward) and measured the influence of both on the speed of responding. The findings strongly suggest that post action selection, responding is influenced only by pure effectiveness, as assessed by the motor system; thus, at these stages responding is not sensitive to abstract representations of the value of a response (e.g., monetary value). We discuss the benefit of distinguishing between these two necessary aspects of adaptive behavior namely, fine-tuning of motor-control and striving for desired outcomes. Finally, we embed the findings in the recently proposed Control-based response selection (CBRS) framework and elaborate on its potential for understanding motor-learning processes in developing infants.

## Introduction

By definition, human behavior is strengthened by reinforcers^1,2^: Valued outcomes increase the vigor and frequency of actions that have produced them. As those reinforcing indices have been repeatedly linked to the value or worth of outcomes, it came as a surprise that substantial body of recent research found that responses can also be reinforced by neutral outcomes. Specifically, the mere fact that a response leads to a mundane perceptual change in the environment has been repeatedly demonstrated to be enough to increase the speed and to some degree, the frequency of that response^3–7^.

These findings could be taken as evidence that action-effectiveness generates value and hence, by making the previously neutral perceptual effect valuable, influences responding through the same mechanism as established reinforcers - such as monetary rewards - do. Yet, some empirical evidence as well as theory suggests that action effectiveness and ‘classic’ reinforcers operate through somewhat different routes. In the present paper, we test whether monetary rewards as classic reinforcers motivate behavior in the same way as action effectiveness does.

### Action effectiveness as a reinforcer

The reinforcing power of mere action effectiveness was first demonstrated in our Effect Motivation task^3,4^. In this task, participants have to “stamp” colored circles falling down from the top of a computer screen randomly in one of four lanes, using four different response keys. Results show that responses where significantly speeded up if key presses were immediately followed by a behavioral change, such as the circle changing color. Under some conditions, such direct action-effects also increased the frequency of selecting the relevant responses. In short, in every way, action effectiveness acted as a reinforcer of behavior.

However, some findings suggest that action effectiveness in the Effect Motivation Task does not reinforce behavior in the same way as classic reinforces. For example, while behavior-contingent reinforcement (e.g., token reinforcement^8^), has been shown to successfully strengthen behavior, we^3^ (Exp 2) have found that repeatedly attaining game points for correct performance – – did not lead to faster responding above and beyond mere action effects. Similar findings were obtained in a recent study^6^, in which an affirmation of participants correct responding (a well-known reinforcer) that wasn’t spatially linked to the response, failed to facilitate response speed. Finally, in yet another study^7^ participants suffering from major depression (MDD), which is associated with deficient reward processing^9^, displayed fully intact sensitivity to effectiveness feedback. Specifically, their response speed was facilitated by own-action effects at least as strongly as it was in healthy participants.

These anecdotal findings demonstrate that although effectiveness in the Effect Motivation Task clearly reinforces behavior, it may operate through a somewhat different route than ‘classic’ reinforcers do. Given that this would suggest a modification of the current understanding of the ‘reward system’, the present research further explores this possible dissociation by directly testing whether the value of outcomes reinforces responses at the motor level as effectiveness was found to.

### Motivation and response selection

Much of current work on how motivation affects response-selection focuses on the brain’s ‘reward-system’. According to current models of how this system relates to response selection, the striatum — a sub-cortical area which is a key part of the basal-ganglia — is important for representing the predicted worth of specific actions and influencing the response selection process accordingly^10–14^. For instance, a positive or desired outcome (such as money or food) that is contingent on one’s action is represented with its predicted value related to the specific action that it is contingent upon^13^. Given the perception of an ‘appetitive stimulus’ (a stimulus which was found to be reinforcing upon consumption), the response that is represented with a higher predicted value is more likely to be selected among other response options^13^.

Given such a mechanism of behavioral control, one route for the modification of responding – an increase in the frequency of responding^15–18^– can be explained by the modulation of (response) selection by predicted *value*. As it is a consensus that the actions of animals – that is the what and whether of doing – are selected by their relationship with predicted value/worth or a computation in which predicted value figures in; the specific question we ask here is whether predicted value also influences the lower levels of selection, the specific movement through which an (abstract) action is manifested.

Theoretically, as we further develop below, while modulation of responding by effectiveness is driven by a match between predicted and perceived outcomes (i.e., the output of the ‘comparator model’ previously proposed to explain human’s ‘sense of agency’^19^); modulation of responding by value is seemingly driven by various assessments of utility or goal-relevance^20–22^.

### The control-based response selection (CBRS) framework

Focusing on the modulation of a specific movement is based on the findings suggesting that response-contingent effects also facilitate response speed when response selection is fully specified^18^. The findings of our previous studies are summarized in a working-model named the Control-based response selection (CBRS) framework^5,6,23^. Specifically, the CBRS framework places the effects of pure (to differentiate from the typical utility-based notion of effectiveness) response-effectiveness as affecting lower levels of response selection. More specifically, the lower levels (which are part and parcel of the motor-system) are the ones which occur after a response is fully specified at a relatively abstract level (i.e. through the specification of a motor goal and the operation of an inverse model to attain that goal^24–26^). According to the CBRS framework, this post action-selection modulation of responding depends on the outputs of a relatively simple mechanism that the brain uses to evaluate the effectiveness of its motor programs – the Comparator model^27,28^.

A key principle of the CBRS framework is that different levels of response selection are sensitive to different inputs. The more abstract level at which action is selected (the selection of what/when/whether to perform an action; such as ‘get the coffee mug’) may be sensitive to the Comparator model’s output and also receive inputs such as goals, utility-like calculations and the results of the representation of the abstract requirements of the task at hand (such as ‘respond rapidly’).

Conversely, the *sole* input to the ‘lower’ levels – the specific motor programs that are to be activated – are the outputs of multiple comparators as specified by the optimal control theory of motor control beyond the initial operation of an inverse model^24–26^ (For a dissenting position^29^).

In our work, we have focused on subpart of the above comparators; specifically, on the modulation of the motor program by the so called ‘comparator model’ (or ‘third comparator’) –which compares the reafferent (incoming or sensed) stimulation with the sensory-motor prediction generated by the efference copy^24^ (although it has been recently argued that sensory-motor predictions can also be generated in other ways^30^)

The primary contribution of the CBRS framework is in linking between the above, motor system-based assessment of a response’s effectiveness (enabled by the machinery specified in the ‘comparator model’) and the parameters this assessment is sensitive to with their potential to modulate different levels of response selection (higher vs. lower).

Of most relevance to the current study, is that the CBRS framework is consistent with the prediction that the lower levels of *motor programing* will not be sensitive to classic reinforcers such as the monetary value associated with a specific response if these influence only the initial selection of the motor program (the inverse model^24^; see also^31^). This is what the current study set out to test.

## The Current Study

Here we adapted a task repeatedly shown to generate response time (RT) facilitation by pure effectiveness feedback to test whether monetary value will produce the same effect. Importantly, in this task, speeding up of RT by own action-effects was seen even though the variability during the action selection phase (‘inverse model’) was minimized by cueing the required response.

First, we tested whether the speed of response selection in humans is sensitive to different expected positive outcome values (i.e. substantial and negligible monetary values) when pure response-effectiveness is provided. (Experiments 1a and 1b). As the pattern of results was inconclusive, we conducted two further experiments (Experiments 2a and 2b) retesting the critical conditions. This presented a clearer answer to the above questions. Experiment 3 was conducted to attempt and replicate these findings in a different task which better controls for speed accuracy tradeoff (a potential concern in the previous experiments). Finally, Experiment 4 was conducted to provide a comprehensive examination of the above question by directly examining whether modulation of responding in humans, post action selection, is sensitive to value (e.g., monetary values) both with and without pure effectiveness feedback. In all experiments we applied a between-subject design to avoid carryover effects (i.e., a potential change in the value of the monetary reward and\or the value of pure response-effectiveness because of gaining smaller\larger sums in the previous block of trials). Given variation in the sample size per condition between experiments and to quantify the strength of evidence for our conclusions, we used Bayes factor for all critical analyses.

## Results

### Experiment 1a & 1b: The influence of monetary value on response selection when pure effectiveness-feedback is provided

**Figure 1:**
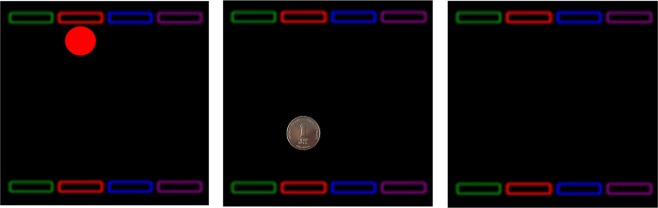
An illustration of a trial in the Substantial Monetary Gain with pure response-effectiveness feedback condition. Participants are instructed to press a spatially and color-coded key on a standard keyboard when they first see the descending cue (left box). Immediately after an appropriate response, the circle cue changes to a one NIS coin for 200 ms (middle box) and disappeared until the end of the trial (right box). The 1 NIS coin image depicts a unit of currency issued by the Bank of Israel. This design is copyrighted by the Bank of Israel.

In the Effect Motivation task^3,4^ participants face a computer screen and place their fingers on four designated response keys (‘S’, ‘D’, ‘H’ and ‘J’). On each trial a colored circle (a target) appears in one of four possible horizontal locations at the top of the screen and rapidly descends in a vertical downward path. Participants are instructed to ‘stamp’ the circles as they appear on the screen by pressing the relevant spatially coded key. In Experiment 1a, participants were randomly assigned to one of three between-subject conditions: a Substantial Monetary Gain condition in which each appropriate response immediately affected the target circle that changed into an Israeli Shekel coin (~0.3 US Dollars) for 200 ms, followed by its disappearance until a new trial begun (See Fig. 1 for an illustration); a Negligible Monetary Gain condition in which the target circle-cue changed into an Israeli coin called an Agora (0.003 US Dollars or ~0.3 US cents) which although familiar to participants is now out of circulation; and a *No-Effect*/No-Gain condition in which responses changed nothing on the screen and the target circle continued its descent to the bottom of the game window. Crucially, in the monetary effect conditions, participants were explicitly told that at the end of the experiment they would receive the sum value of the coins that will appear on the screen during the task.

In *Experiment 1b* we used three additional conditions (six conditions over all): a *Substantial Monetary No-Gain* condition in which the monetary gain was not mentioned in the instructions and thus, participants did not expect to actually receive the many Israeli Shekel coins that appeared, conditional on their responses; a *Negligible Monetary No-Gain* condition that was identical to the Negligible Monetary Gain condition except that here too, the possibility of an additional monetary gain was not mentioned to participants; thus, participants in this condition did not expect to actually receive the multiple (worthless) Agora coins that appeared, conditional on their responses; for comparison purposes, we also added a (standard) *Effect* condition in which the target-cue changed its color to white for 200 ms immediately after participants’ (correct) response which was experienced as a ‘flash’.

Based on the theoretical and empirically established links between tangible rewards and response selection^11–14^, the consensual prediction is that given that the degree of pure effectiveness is identical in the four different monetary conditions (i.e., in all conditions a perceptual effect immediately follows responses) — participants’ responses would be faster in the Substantial monetary condition compared to the Negligible monetary condition *but only if they expect to gain the money*; critically, when participants do not think there is real money to be gained, we expected the facilitation in response selection due to variation in receiving own-action effects to reappear (compared to the No-effect condition^3–7,23^).

**Figure 2:**
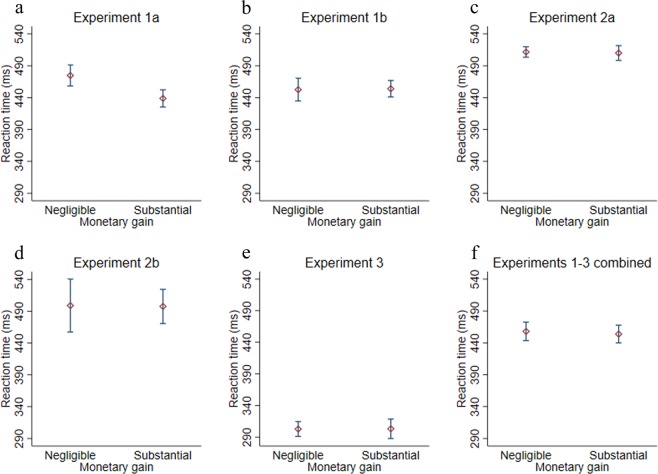
Experiments 1a-3: The impact of monetary value on the speed of response-selection. (**a**): Experiment 1a. (**b**): Experiment 1b. (**c**): Experiment 2a. (**d**): Experiment 2b. (**e**): Experiment 3. (**f**): all five experiments combined. Error bars depict 95% confidence intervals.

Before analyzing the data we applied a similar filtering procedure as in previous experiments using this task in the following order: participants with less than 85% correct responses (Exp.1a: one participant; Exp.1b: 4 participants), incorrect responses (Exp.1a: 324/10620 = ~3%; Exp. 1b: 1364/ 27720 = ~5%), responses that were either above 700 ms or below 200 ms (Exp. 1a: 517/10620 = ~5%; Exp. 1b: 524/ 27720 = ~2%), and trials that deviated from their condition’s mean by at least 2 standard deviations in their mean reaction time (Exp. 1a: 451/10620 = ~4%; Exp.1b: 1134/ 27720 = ~4%). Total filtration rate was 13.5% of the raw data. Filtered responses were removed and not analyzed further. To statistically test our predictions, we used a one-way ANOVA and additional two-tailed between-subject t-tests, if not specified otherwise. Furthermore, to quantify the degree that the data supported the critical hypotheses (the null or the alternative), we also conducted non-directional Bayesian t-tests using JASP^32^ using the default Cauchy prior (width = 0.707) for all critical comparisons and report the Bayes Factor (BF). For better estimation of the parameters we present the analyses on a pooled data from both Exp. 1a and 1b (see Fig. 2 and Supplemental Materials for separate analyses for the individual experiments).

As a behavioral proxy for the speed of response selection, we analyzed participants’ reaction times (RT^3–7,15,16^). A one-way ANOVA with Feedback type as a between-participant factor with six levels on participants’ mean reaction time was statistically significant [F(5, 201) = 4.24, Root MS = 35.35, *p* < 0.01]. Next, we tested whether, when monetary gains are not expected, reaction time would be sensitive to response-effectiveness (operationalized as an immediate response-contingent effect). Critically, reaction times were significantly shorter in the Negligible monetary no-gain (M = 451, SD = 31) and (only in a one-tailed comparison) in the Substantial monetary no-gain conditions (M = 458, SD = 23), compared to the No-effect condition (M = 474, SD = 43) [t64 = 2.29, p = 0.02, BF10 = 2.3 (inconclusive), CI_95_ (3, 43), d = 0.61] [one tailed t66 = 1.73, p = 0.04, BF10 = 1.7 (inconclusive), CI_95_ (−2, 34), d = 0.46], correspondingly; and there was no significant difference between the Negligible monetary no-gain and the Substantial monetary no-gain conditions [t48 = 0.91, p = 0.36, BF10 = 0.4 (near substantial support for the null), CI_95_ (−8, 22), d = 0.25]. In addition, replicating previous findings^12,14,23^, RT was robustly shorter in the Effect (M = 440, SD = 33) compared to the No-effect condition [*t*_66_ = 3.41, *p* < 0.01, BF_10_ = 13.39 (conclusive), CI_95_ (14, 53), d = 0.88]. These findings are consistent only with the influence of pure response-effectiveness on the speed of response selection.

When participants expected to gain the money, it seemed that RT tended to be shorter in the Substantial monetary gain condition (*M* = 447, *SD* = 31) compared to the Negligible monetary gain (*M* = 462, *SD* = 39) condition [*t*_87_ = 2.06, p = 0.04, BF_10_ = 1.4 (inconclusive), CI_95_ (0.54, 30), d = 0.43].

Thus, the findings from both Experiments 1a and 1b demonstrate once again the robustness of the effect of pure effectiveness feedback on the speed of response selection. However, given the inconclusive Bayes Factors (0.3 < BF < 3) and crucially, the opposite conclusion stemming from the pattern observed in Exp.1a and Exp.1b for this contrast, we are unable to conclude on the basis of the first two experiments alone whether RT is sensitive to the monetary value of the outcome [see Supplemental Materials for analyses of the individual experiments and for further exploration of the data from Exp. 1b (which was motivated by our prior, not sufficiently thought out and naive belief that there should be an effect of monetary value cum reward on RT). Specifically, we analyzed only Hebrew native speaker participants who reported to believe they would receive the monetary gain. Notably, this exploration ultimately failed to yield a reliable effect of monetary value on RT)].

### Experiments 2a and 2b: The influence of monetary value on response selection when pure effectiveness-feedback is provided: a replication

Experiment 2a and 2b were conducted to further investigate whether and how would both monetary value and response-effectiveness influence the speed of response selection using the same task as in Exp. 1, but with only the three critical conditions (Substantial monetary gain, Negligible monetary gain and No-effect conditions). Furthermore, Experiments 2a and 2b were conducted at different academic institutions (a research university and a college) to increase the generalizability of the results. In addition, the experimenter verbally emphasized to the participants that they will receive a real monetary reward proportional to the sum of coins they would gain in the task. This minimized any ambiguity that may have existed regarding the actual compensation policy.

Before analyzing the data, the following filter procedure was applied: participants with less than 85% correct responses (Exp. 2a: one participant, Exp.2b: 2 participants), incorrect responses (Exp. 2a: 896/17100 = ~5%; Exp. 2b: 384/8100 = ~5%), responses that were either above 700 ms or below 200 ms (Exp. 2a: 727/17100 = ~4%; Exp. 2b: 681/8100 = ~8%), and trials that deviated from their condition’s mean RT by at least 2 standard deviations (Exp. 2a: 555/17100 = ~3%; Exp. 2b: 259/8100 = ~3%). Total filtration rate was 16% of the raw data. Filtered responses were removed and not analyzed further. For better estimation of the parameters we present the analyses on pooled data from both Exp. 2a and 2b. All tests were two-tailed (see Fig. 2 and Supplemental Materials for a separate analysis for each experiment).

Similar to the former experiments, evidence for *no difference* in participants’ RT was found between the Substantial (M = 506, SD = 41) and Negligible (M = 508, SD = 39) monetary gain conditions [*t*_(121)_=0.31, p = 0.75, BF_10_ = 0.20 (conclusive), CI_95_%(−12, 16), *d* = 0.04]. In addition, RT in both Substantial [*t*_(71)_=2.90, p < 0.01, BF_10_ = 8.23 (conclusive), CI_95%_(12, 68), *d* = 0.77] and Negligible [*t*_(74)_=3.17, p < 0.01, BF_10_ = 15.98 (conclusive), CI_95_ (16, 70), *d* = 0.82] monetary conditions were significantly *slower* than the No-effect condition (M = 465, SD = 63).

A post-hoc exploration of this unpredicted pattern revealed a speed-accuracy tradeoff in Exp. 2a in the Substantial monetary gain condition (*r* = 0.46, *p* < 0.01), but not in the Negligible monetary gain condition (*r* = −0.02, *p* = 0.87) and in Exp. 2b in both monetary gain conditions (Substantial: *r* = 0.55, *p* < 0.01; Negligible: *r* = 0.81, *p* < 0.01; No-effect: *r* = 0.31, *p* = 0.29). Following these findings, we returned to explore whether a speed-accuracy tradeoff also existed in the 2 previous experiments and found an inconsistent pattern between Exp. 2a and 2b and between Exp.1a (Substantial: r = 0.38, p = 0.09; Negligible: r = −0.46, p = 0.03; No-effect: r = 0.13, p = 0.61) and Exp. 1b (Substantial: r = −0.24, p = 0.22; Negligible: r = 0.13, p = 0.54; Effect: *r* = 0.01, *p* = 0.93; No-effect: *r* = 0.32, *p* = 0.11).

Although the pattern does not explain why RT did not differ between the Substantial and the Negligible monetary conditions, it does suggest that speed-accuracy tradeoff may occur in conditions in which an outcome (vs a ‘pure’ effectiveness) is available and hence, that outcomes may activate different (more ‘controlled’ or ‘strategic’) processes than when only pure effectiveness is involved^2,3^ This is consistent with reward being able to influence the ‘high’ levels of action selection – up to and including the generation of an inverse model. A methodological note, it only makes sense to use a measure that combines response time and proportion correct when they are assumed to tap into the same processes in a specific context^33^ – an assumption we are not comfortable to adopt at this stage.

To better control for a speed-accuracy tradeoff, we conducted Experiment 3 in which we used a different task where participants choose freely and randomly their response on each trial^4^. Importantly, the task strongly suggests that no correct response exists and hence enables us to examine RT differences as a function of varying monetary values by minimizing speed-accuracy tradeoff.

### Experiment 3: The influence of monetary value on responding when pure effectiveness-feedback is provided: a free-choice task

To minimize the threat that a speed-accuracy tradeoff may contaminate or otherwise mask the facilitating effect of monetary gain on the speed of response selection, we used a modified version of the Effect-Motivation Free-Choice task^4^. In the task, participants are instructed to randomly select one of four possible responses whenever a cue appears at the center of the screen. Since there is no requirement for (or even a definition of what would count as) a correct response in this task, there is no or at least, substantially less incentive for trading off speed for accuracy– increasing the probability of detecting an effect of monetary value on the speed of response-selection, if such an effect exists.

Before analyzing the data, the following filters were applied: missing trials (188/12060 = 1%), responses that were either above 700 ms or below 200 ms (2073/12060 = ~17%) and trials that deviated from their condition’s mean RT by at least 2 standard deviations (582/12060 = ~5%). Total filtration rate was 23% of raw data. Filtered responses were removed and not analyzed further. All tests were two tailed.

Again, evidence for *no difference* in participants’ RT was found between the Substantial (M = 303, SD = 32) and Negligible (M = 303, SD = 26) monetary gain conditions [*t*_(39)_=0.03, p = 0.97, BF10 = 0.30 (conclusive), CI_95_(18, −18), d = 0.01; see Fig. 2e]. In addition, RT was only nominally shorter in both the Negligible [*t*_(46)_ = 1.64, p = 0.10, BF_10_ = 0.85 (inconclusive), CI_95_(−3, 31), *d* = 0.48] and the Substantial [t(43) = 1.42, *p* = 0.16, BF_10_ = 0.67 (inconclusive), CI_95_(−5, 33), *d* = 0.43] monetary gain conditions compared to the No-effect condition (M = 317, SD = 33). The combined monetary conditions were significantly faster than the No-effect condition, but only in a one-tailed test [one-tailed *t*_(65)_ = 1.85, *p* = 0.03, BF_10_ = 2.07 (inconclusive), CI_95_(−1, 29), d = 0.45] and the raw effect size was smaller than previously observed for ‘pure’ effectiveness (which was ~25 ms).

#### Analyzing the effect of monetary value on the speed of response-selection on pooled data from all five experiments

Given the variation between studies and filtration rates, to get a better handle on whether the speed of response selection is sensitive to the response’s value, we conducted the following analyses on the pooled data from all five experiments. Specifically, we regressed RT on monetary value (Substantial vs. Negligible) while controlling the variability explained by the individual experiments (df_model_ = 2, df_residual_ = 250, Root MSE = 72.62, R^2^ = 0.19). We found that the monetary value of action’s outcome did not predict RT [p = 0.26, β = 0.06, CI_95_(−7, 28); see also Fig. 2f]. Importantly, a Bayesian analysis provides conclusive support for the null (BF_01_ = 3.93). Thus, the full pattern of findings leads us to conclude that the post inverse model (action selection) stages of a response selection are insensitive to the monetary value of the outcome *while being responsive to a response’s pure effectiveness*.

### Experiment 4: The influence of monetary value on response selection both with and without pure effectiveness-feedback

Experiment 4 was conducted to test whether this pattern replicates and address two potential limitations of Experiments 1–3 (We are deeply grateful for an anonymous reviewer for proposing this experiment). Specifically, in all previous experiments monetary gain was always combined with pure effectiveness-feedback (an action-effect) which may have overshadowed any potential effect of monetary gain on the speed of response-selection (due to, for example, a ceiling effect of speed). In addition, the monetary value in the Negligible monetary conditions (1 Agora coin) is now out of circulation and perhaps its monetary value is ambiguous to some of the participants, resulting in relatively large individual differences in the represented value of the coin. In Experiment 4, we addressed both issues by examining the sensitivity of the speed of response selection to different monetary values both with and without pure effectiveness feedback (a response-contingent effect). In addition, for the Negligible monetary condition – instead of 1 Agora coins, we used a 10 Agorot coin as negligible monetary reward; which is at the time of running this study, the lowest value Israeli coin in circulation (~0.03$; see Table 1 for the experimental design).

**Table 1:** **Experiment 4** – Experimental design.

**Between-subject Conditions**	**Within-subject block**	**Within-subject block**
Substantial Monetary Gain	With pure effectiveness feedback	Without pure effectiveness feedback
Negligible Monetary Gain	With pure effectiveness feedback	Without pure effectiveness feedback
No-Effect	With monetary bonus	Without monetary bonus

Based on the findings above, we predicted that RT in this task will be sensitive to a response’s effectiveness but not to the monetary value expected from executing it (This experiment was pre-registered: https://osf.io/v8gxj/).

Before analyzing the data we applied the same filtering procedure as in previous experiments using this task in the following order: participants with less than 85% correct responses (7 participants), incorrect responses (1697/34200 = ~5%), responses that were either above 700 ms or below 200 ms (576/34200 = ~2%), and trials that deviated from their condition’s mean by at least 2 standard deviations in their mean reaction time (1269/34200 = ~4%). Total filtration rate was 17% of raw data. Filtered responses were removed and not analyzed further. All tests were two-tailed.

**Figure 3:**
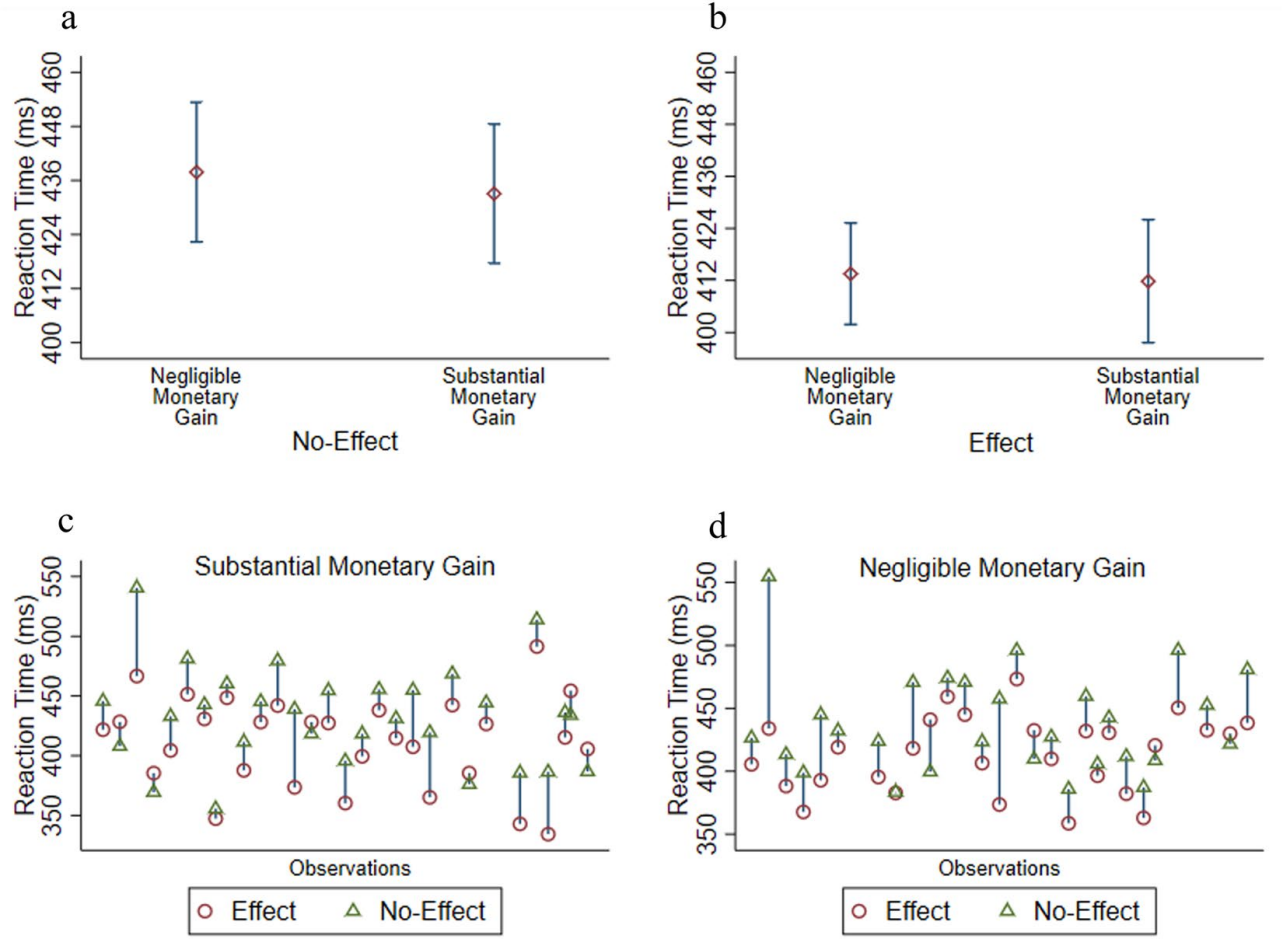
Experiment 4 - The impact of pure effectiveness-feedback and monetary value on the speed of response-selection. (**a**) presents the difference in mean RT between monetary values when pure effectiveness-feedback is not provided. Error bars depict 95% confidence intervals. (**b**) presents the difference in mean RT between monetary values when pure effectiveness-feedback is provided. Error bars depict 95% confidence intervals. (**c**) present differences in RT for each participant in the Substantial Monetary Gain condition, in blocks with and without pure effectiveness-feedback. (**d**) presents differences in RT for each participant in the Negligible Monetary Gain condition, in blocks with and without pure effectiveness-feedback.

First, in condition blocks with monetary gain and without pure effectiveness-feedback, RT was not different between the Negligible (M = 437, SD = 39) and the Substantial (M = 433, SD = 41) monetary conditions [*t*_56_ = 0.44, *p* = 0.65, CI_95_(−16, 26), *d* = 0.09; See Fig. 3a]. A Bayesian non-directional t-test provided a conclusive support for the null (BF_01_ = 3.45). In condition blocks *with* pure effectiveness feedback there was also no significant difference in RT between the Negligible (M = 413, SD = 30) and the Substantial (M = 411, SD = 38) monetary conditions [*t*_56_ = 0.18, *p* = 0.85, CI_95_(−16, 19), *d* = 0.05; See Fig. 3b]. Here too, a Bayesian non-directional t-test provided a conclusive support for the null (BF_01_ = 3.7).

Next, we examined the effect of response-effectiveness on RT for both Substantial and Negligible monetary values. A paired-sample t-test revealed that for both Substantial [*t*_29_ = 4.75, *p* < 0.01, CI_95_(12, 30), *d* = 0.86; See Fig. 3c] and Negligible [*t*_24_ = 4.18, *p* < 0.01, CI_95_(12, 36), *d* = 0.79; See Fig. 3d] monetary values, RT was shorter when pure effectiveness feedback was given (Substantial: M = 411, SD = 37; Negligible: M = 413, SD = 30) compared to condition blocks without pure effectiveness feedback (Substantial: M = 433, SD = 41; Negligible: M = 437, SD = 39). A Bayesian non-directional paired-sample t-test provided strong support for the effect of response-effectiveness on RT in both Substantial (BF_10_ = 479.6) and Negligible (BF_10_ = 107.2) monetary conditions. Note that this, again, shows that it is not the case that the current task is merely insensitive to capture differences in response times. Rather, it is monetary value that is again found to be ineffective at generating such differences.

Finally, there was *no difference* in RT between the No-effect with (M = 432, SD = 38) and without (M = 428, SD = 40) non-contingent monetary bonus [*t*_29_ = 0.98, *p* = 0.33, BF_10_ = 0.30 (conclusive), CI_95_, (−3, 10), *d* = 0.17].

## Discussion

The recent evidence for the motivating impact of response-effectiveness^2–7^ and the extensive literature demonstrating motivational effects of attaining and observing desired outcomes on responding (e.g.^13,14^,), raise the fundamental question of whether these reinforcers act through the same routes.

Previous work documented that the magnitude of the expected reward seemingly affected the speed of responding on the scale of seconds (e.g., the Crespi effect^34^). Here we measured human participants’ response speed at the millisecond (ms) scale which is more appropriate for detecting modulation of motor processing stages^23,35^.

While we naively expected that pure effectiveness and an outcome’s value would additively facilitate response selection, this was not supported by the data. Specifically, we repeatedly found that pure response-effectiveness (operationalized as the existence of an immediate action-contingent perceptual effect), but not its expected value (operationalized as the response-contingent monetary value), influences the speed of response selection.

### Different strokes for different folks: selection of actions vs selection and/or modulation of lower-level motor programs’ parameters

Ironically, the pattern we found (but not our initial prediction) is consistent with our own working-model – the Control-based response selection (CBRS) framework^5,6,23^. According to this framework, the levels following the translation of a desired end point of a movement to motor programs (using an inverse model) are detached from the value of such desired end states (c.f.^36^,) and are presumably monitored and modulated through multiple comparators.

At these levels, modulations operates ‘blindly’ in the following senses: (1) it is sensitive only to parameters that are computed by or otherwise available to these low-level post-action selection parts of the motor-system such as: learned action-effect conditional probability, the temporal contiguity of the effect with the action and the spatial predictability of the effects^3–7^ and (2) it is seemingly ‘blind’ to cognitive expectations about the task such as the value of the outcome^37,38^; but see^39^. Note that the ability of such post action-selection stages (and their relevant parameters) to modulate further action goals (beyond motor specifications) is not fully known; yet, it is conceivable that post action selection stages are limited and are dependent on the initial generation of an inverse model (i.e. motor goal-based action selection). As previous and the current findings imply, the motor goal generated by an inverse model includes no representation of outcome values.

It is worth noting that such post action-selection stages are not akin to the so called ‘dorsal route’ of action selection^40^, as (at least part of) such route was demonstrated to circumvents value expectancies^41,42^.

While previous studies have seemingly documented the effect of desired (or ‘positively valenced’) outcomes on response speed (e.g.^43^,), it is not clear which stage of the response-selection (e.g., the selection of the effector or specific response parameters) was affected by the outcome’s value. In fact, there are previous demonstrations suggesting that monetary reward has a differential influence on different stages of response selection. For instance, Mir *et al*.^44^, showed that monetary reward facilitated RT only when participants were able to plan their response in full (i.e. when they knew in advance which response to prepare). Similar to the current tasks, when participants were not able to prepare their responses in advance (the uCRT condition in Mir *et al*.^44^,), RT was not influenced by the expected reward.

The current work fits well with Mir *et al*.s’^44^, findings and strongly suggests that expected outcome value (as far as it is captured by the response’s monetary outcome) has no influence over post action selection stages, and regardless of whether expected outcome value was manipulated within^44^ or between-subjects (the current study). Note that beyond further establishing the above conclusion, the current study extends Mir *et al*.^44^, findings by empirically dissociating between the effects of the two different types of rewards on advanced stages of response-selection.

Note that the CBRS framework allows for an expected outcome value to modulate response speed (e.g., through the decision how quickly to perform an action, through the decision which course of action to perform). These of course involve top-down processes regarding the cost and benefit of performing for example a quick response (e.g., response vigor^18,41,45,46^). Crucially, these types of decisions are resolved by the generation of an inverse model - the point of which such considerations apparently lack influence.

Importantly, RT in the current task was previously shown to be unassociated with participants’ perceived effort and perceived speed^4,5^ and, in the current study, with the outcome value of the response. Conversely, response-effectiveness facilitated RT even after statistically controlling for participants’ attentional engagement in the task^4^. Future studies may modify the task in a way that encourages vigorous responding while examining the distinct contribution of both outcome value and pure response effectiveness on overall RT; for example, by using distinct response cues for different monetary value trials, letting participants affect RT through repeated selection of an action, or using a challenging short response-window linked to the trial’s outcome (without giving a perceptual effect following a response). Our CBRS-based prediction for the latter experiment would be that participants will tradeoff speed for accuracy – as an intentional attempt to maximize reward. Therefore, participants in the high-value condition may be as fast as their counterparts in the pure effect condition but will make significantly more errors.

### Implications and future directions

#### Two types of being effective – a mechanistic implementation

The discovery that different reinforcers (action-effectiveness and outcome) may affect responding through different levels of response selection can be also understood as facilitating two distinct aspects of adaptive behavior namely, optimal motor-control and goal pursuit. More specifically, we suggest that rather than these two dimensions of ‘being effective’^47^ being simply integrated, for example at the level of the reward signal, they modulate different stages of the response. Mechanistically, the implementation of this idea requires little modification of existing ideas. The two types of information that are required to credit a motor plan for ‘outcome effectiveness’ (attaining a desired outcome) and for ‘pure (control) effectiveness’ are already separated in both the optimal control models of motor control^48^ and their offshoot – the comparator model^27,28^. The functions of the inverse model, as described above is separated from the working of the comparators: comparator 1 (comparison between goal and current state) and 3 (comparison between efference copy-driven sensory prediction and reafferent stimulation), correspondingly. Previous versions of the CBRS framework already include an element that reinforces purely effective motor-plans (i.e. regardless of outcomes) as well as the differentiation between representations of actions and motor responses (for the latest version^6^); in line with current frameworks of action selection (e.g.^49^,). Thus, the only modification required by the current data would be to explicitly state a link between the working of the inverse model and selection at the abstract (action) level (e.g.^50^).

### Competing frameworks

Proponents of a different theoretical framework, the ideomotor theory (for a review^51^) and its modern version namely, the Theory of event coding (TEC^52^), have also proposed to differentiate between control (action-effect congruency) and ‘valenced’ aspects of an action^53^; see also^54^,). Specifically, Eder *et al*.^55^, suggested that while the representation of the expected action-contingent effect is enough to activate the motor code associated with the effect, an additional process may inhibit or facilitate the execution of the action based on its expected valence.

Notably and similar to our results, they too failed to find a facilitation effect of actions associated with positive versus negative outcomes. Moreover, participants’ response speed was facilitated when their action produced an effect, but only when the effect entailed the same valence (positive or negative) expected given a previous acquisition phase (see also^53^).

While the model proposed by Eder *et al*.^55^ suggests that the influence of action-control and expected valence on action selection is additive, as stipulated above; in the CRBS framework, levels of response-selection are differentiated to suggest that *selection at its lower levels is insensitive to outcome expectancies and beliefs (such as one’s expectations regarding the valence of action’s outcome)*. As stated above, the CBRS framework allows for an action’s outcome-value to have a strong influence on choosing between alternative optional actions (for example) but not on the exact manner of its execution. This is the pattern that was supported by the current data.

Another framework that is relevant to the current findings focuses on the incentive salience of the conditional stimulus (here the response-cue) which may reinforce behavior on its own, reflecting the ‘wanting’ aspects of the reward-system^56^. Regarding the current findings, it is theoretically possible that cues signaling an opportunity to exercise control (as generating action-effectiveness feedback; compared to no-feedback) have stronger incentive salience. However, if this was the key mechanism, we would expect it to also operate in the case of the monetary value of the action-effect. But the results do not support this possibility. As elaborated above, according to our CBRS framework, such mechanism operates at a higher level of action-selection.

Clearly, further work is necessary to fully elucidate the computational and neural mechanisms underlying the influence of action-effectiveness on response selection and the potential combined influence of pure effectiveness and outcome values on action selection (e.g., the selection of “what/when/whether to do”^57^; see also^58^); but given our previous findings showing a connection between abstract knowledge and high-level selection of actions^4^ it is likely that (in humans at least) abstract knowledge of the relationship between rewards and actions, are necessary for the objective rewards to promote intentional actions^59,60^.

Finally, these findings call for developmental work, as the properties of the relatively unexplored type of motivator — control-effectiveness —its tight connection to the motor system and its apparent insensitivity to pursuing desired outcomes, suggest that it may be important for very early stages of motor control and, potentially action learning in the developing child^61,62^. Learning own-action contingencies in such a manner may also serve as a rudimentary, motor system-based, form of causal learning of the affordances of one’s both internal and external environment^63,64^.

## Methods

### Experiment 1a and 1b: the influence of monetary value on response selection when pure effectiveness-feedback is provided

#### Participants

**Experiment 1a**. Fifty-nine undergraduate students [41 females, Age (*M* = 25.35, *SD* = 3.8)] from the University of Haifa participated in the experiment in exchange for course credit or 20 New Israeli Shekels (~$5.5). **Experiment 1b**. One hundred and fifty-four undergraduate students at the University of Haifa who did not participate in Experiment 1a were recruited from the same pool [99 females, Age (*M* = 24.37, *SD* = 4.28)] in exchange for course credit or 20 Shekels (~$5.5). Participants who were assigned to conditions in which they could earn additional payment, were informed about their assignment only when receiving the task’s instructions. All participants in the following experiments signed informed consent. The study was approved by the departmental ethics committee of the University of Haifa (Israel) and carried out in accordance with the approved guidelines.

#### Stimuli and procedure

We used a modified version of the Effect Motivation task^2–4^, the cited work used participants’ reaction times as an index of the speed of response selection. Participants sat in individual rooms with dimmed lighting, in front of a computer screen and placed their fingers on four designated response keys (‘S’, ‘D’, ‘H’ and ‘J’) on a standard PC keyboard. A trial began with the presentation of a colored circle (a target) in one of four possible horizontal locations at the top of the screen; on appearance, the circle rapidly descended in a vertical downward path. Participants were instructed to appropriately ‘stamp’ the circles as they appear on the screen by pressing the relevant spatially coded key.

**In Experiment 1a**, participants were randomly assigned to one of three between-subject conditions: a *Substantial Monetary Gain* condition in which each appropriate response immediately ‘affected’ the target circle that changed into an Israeli Shekel coin (~0.3 US Dollars) for 200 ms, followed by its disappearance until a new trial began; a *Negligible Monetary Gain* condition in which the target circle-cue changed into an Israeli coin called an Agora (0.003 US Dollars or ~0.3 of a US cent) which, although familiar to the participants, is now out of circulation; and a *No-Effect*/*No-Gain* condition in which responses changed nothing on the screen and the target circle continued its descent to the bottom of the game window. Crucially, in the monetary effect conditions, participants were explicitly told that at the end of the experiment they would receive the sum value of the coins that had appeared on the screen during the task.

**In Experiment 1b**, we used three additional conditions (six conditions overall): a *Substantial Monetary No-Gain* condition in which the monetary gain was not mentioned in the instructions and thus, participants did not expect to actually receive the many Israeli Shekel coins that appeared, conditional on their responses; a *Negligible Monetary No-Gain* condition that was identical to the Negligible Monetary Gain condition except that here too, the possibility of an additional monetary gain was not mentioned to the participants; thus, participants in this condition did not expect to actually receive the multiple (worthless) Agora coins that appeared, conditional on their responses; for comparison purposes, we also added a (standard) *Effect* condition in which the target-cue changed its color to white for 200 ms immediately after participants’ (correct) response which was experienced as a brief white ‘flash’.

In both experiments, from the appearance of the circle, participants had 1300 ms to respond (until the circle disappeared at the bottom of the screen). A fixed time interval of 2100 ms was maintained between one stimulus’ onset (the appearance of a circle) to another’s (SOA). This held regardless of participants’ reaction time. Participants completed 10 practice trials followed by 180 experimental trials.

After completing the experiment, participants completed a self-report questionnaire about their demographic details and in Exp. 1b they also reported whether they believed they would receive the monetary reward they have gained at the end of the experiment (see Supplemental Materials for the results of participants’ explicit belief which varied). Finally, participants were thanked and were given their compensation for their time and effort plus the additional sum they had gained (indicated below in parenthesis) according to their assigned condition [No effect, Substantial Monetary No-Gain and Negligible Monetary No-Gain conditions: 20 NIS (0), Negligible monetary gain condition 22 NIS (55 cents); and Substantial monetary gain condition: 180 NIS (~44$) in Experiment 1a and 90 NIS (~19$) in Experiment 1b].

### Experiments 2a and 2b: The influence of monetary value on response selection when pure effectiveness-feedback is provided: a replication

#### Participants

**In Experiment 2a***,* ninety-five students [71 females, Age (*M* = 24.89, *SD* = 2.04)] from Tel-Hai Academic College were recruited to participate in the study in exchange for 20 NIS (~5$) or course credit. In **Experiment 2b**, 45 students [28 females, Age (*M* = 23.15, *SD* = 3.19)] from both The University of Haifa and the Technion Institution were recruited to participate in the study in exchange for 20 NIS (~5$) or course credit.

#### Stimuli and procedure

In Experiment 2, we used the same task as in Experiments 1 [Because of a settings error, in Exp. 2a the speed of the circles’ descent was set to 5 pixels per frame and was presented on a screen with a refresh rate of 60 Hertz. This parameter was different from the other experiments in which the speed was set to 8 on a 60 Hertz monitor (or to 4 when the refresh rate of the screen was 120 Hertz). This issue resulted in a slightly slower descent of the circles compared to the other experiments but, crucially was identical for all conditions of this experiment]. In **Experiment 2a**, participants were randomly assigned to one of two between-subject conditions: a *Substantial Monetary Gain* condition and a *Negligible Monetary Gain* condition as in Experiment 1. After completing the experimental task, a computerized self-report questionnaire probed participants’ demographics and the degree they believed they would receive the sum amount of coins that appeared on the screen during the task (see Supplemental Materials for the results of participants’ explicit belief). In **Experiment 2b**, participants were randomly assigned to one of three conditions: one of the two monetary gain conditions as in Exp.2a or to a No-effect condition in which no perceptual effect appeared after their response, exactly as in Exp.1b.

At the end of the experiments, participants were thanked and were given their compensation for their time and effort plus an additional sum (indicated below in parenthesis) according to their assigned condition [No effect: 20 NIS (0), Negligible monetary gain condition 22 NIS (55 cents); and Substantial monetary gain condition: 90 NIS (~19$)]

First, we were interested to examine whether the shorter reaction time in the Substantial Monetary gain compared to the Negligible monetary gain that was found in Exp. 1a replicates. Second, we were interested to examine whether the speed of response-selection is shorter in the Negligible monetary gain compared to the No-effect condition.

### Experiments 3: The influence of monetary value on responding when pure effectiveness-feedback is provided: a free-choice task

*Participants*. Sixty-seven undergraduate students [52 females, Age (*M* = 24.71, *SD* = 4.73)] from the University of Haifa were recruited to participate in the experiment in exchange for course credit or 20 New Israeli Shekels (~$5.5).

#### Stimuli and procedure

The Effect Motivation Free Choice (EMFC) task. In the published version of the EMFC task^4^ each trial begins with the appearance of a red circle (response cue, 53 pixels in diameter) at the center of the game window (dimensions: 413 ×468 pixels). Participants are instructed to voluntarily press one of four PC-keyboard keys (‘S’, ‘D’, ‘H’, ‘J’) each time the red circle appears. Participants are further instructed to take care that the sequence of responses they generate will be as random as possible (i.e., “to avoid any fixed or planned response sequences”). Crucially, whether a response generated an effect was uninformative regarding the attainment of this task-goal. From trial onset (the appearance of the red circle cue), participants had 700 ms to freely select and press one of the four keys. Regardless of response speed, SOA (the time that elapsed between the appearance of one response cue to the appearance of the next) was kept constant at 2000 ms. The task included 10 practice trials and 180 experimental trials.

Exactly as in the previous experiments, participants were randomly assigned to one of three between-subject conditions: a *Substantial Monetary Gain* condition; a *Negligible Monetary Gain* condition and a *No-Effect*/*No-Gain* condition. Crucially, in the two monetary gain conditions, the experimenter further emphasized to the participants that at the end of the experiment they will receive a proportional payment to the sum value of the coins that will appear on the screen during the task (Again, as instructions make clear that any of the 4 possible responses would lead to the effect/outcome – trading speed for accuracy is unnecessary).

### Experiment 4: The influence of monetary value on response selection both with and without pure effectiveness-feedback

#### Participants

Ninety-five undergraduate students [71 females, Age (*M* = 24.22, SD = 4.07)] from the University of Haifa were invited to participate in the experiment in exchange for course credit or 20 New Israeli Shekels (~$5.5).

#### Stimuli and procedure

We used the same Effect-Motivation task as in Exp. 1 and 2 with necessary modifications. Participants were randomly assigned to one of three between-subject conditions of Monetary value (Substantial, Negligible and No-effect conditions) as in the previous experiments. Participants in the two monetary conditions were in fact further assigned to two within-subject conditions of Response-effectiveness (with and without a response-contingent perceptual effect [whether the coin changed or not, according to their monetary gain condition]). The condition was blocked and was administered in a counterbalanced order across participants (see Table 1 and Fig. 1). Apart from using the 10 Agorot (vs Agora) coin, the Monetary + Response effectiveness condition blocks were identical to the previous experiments. In both blocks, participants received identical instructions; namely, that their goal was to ‘stamp’ as many objects as possible by pressing the appropriate, spatially coded, key on the keyboard whenever a cue appears and that they will receive additional pay which is proportional to the sum value of coins that they will gain during the task. Emphasis was given to the value of every response stressing that each appropriate response was worth 10 Agorot (in the Negligible monetary condition) or 1 Shekel (in the Substantial monetary condition) in (proportional) real money which they will receive at the end of the experiment. Crucially, for the Substantial and Negligible without pure effectiveness-feedback condition, no perceptual effect occurred after participants’ response.

Participants assigned to the No-effect condition also performed two blocks in a counterbalanced order (see Table 1). The No-effect without bonus condition block was identical to the No-effect condition in the previous experiments. In the No-effect with bonus condition block, just before beginning to work on this block, participants were informed that they will receive an extra 10 shekels (~3$) bonus for completing this part of the experiment. This monetary bonus was offered to participants regardless of their performance to explore whether non-contingent monetary bonus will increase response speed through mechanisms other than response selection or execution (e.g., by increasing general attentional resources to the task^65^).

After completing the experiment, participants received their compensation for their time and effort plus the additional amounts of money (indicated below in parenthesis) according to their assigned condition [No effect condition: extra 10 NIS (3$), Negligible monetary condition: extra 20 NIS (~6$); and Substantial monetary condition 20 NIS (~ 6$)].

## Supplementary information

SUPPLEMENTARY INFO.

## Data Availability

The datasets generated during the current study are available from the corresponding author on reasonable request.
