# Supplementary material for: The Differential Impact of a Response’s Effectiveness and its Monetary Value on Response-Selection
Source: Sci Rep. 2020 Feb 25;10:3405. doi: 10.1038/s41598-020-60385-9 (PMC7042230; doi:10.1038/s41598-020-60385-9)
Supplement: Supplementary file 1 — SUPPLEMENTARY INFO. [file 41598_2020_60385_MOESM1_ESM.docx]

Supplementary Materials

**The Differential Impact of a Response’s Effectiveness and its Monetary Value on Response-Selection**

**Authors**: N. Karsh, E. Hemed, O. Nafcha, S. Bakbani-Elkayam, R. Custers, &, B. Eitam

**The analyses of the individual experiments (1a-2b) shows why reaching the ultimate conclusion required additional experimentation.**

Experiment 1a

A one-way ANOVA with Feedback type as a between-subject factor with three levels on participants’ mean reaction time was statistically significant [F_(2, 54)_=6.45, Root MS=35.72, *p*<.01]. Specifically, reaction times were shorter in the Substantial monetary gain (M=438, SD=28) compared to both the Negligible monetary gain (M=474, SD=35) and the No-effect (M=473, SD=42) conditions [*t*_38_=3.53, *p*<.01, BF_10_=29.3 (conclusive), CI_95_ (15, 56)*, d* =1.13] [*t*_35_=2.95, *p*<.01, BF_10_=7.87 (conclusive), CI_95_ (10, 59), *d* =.98], correspondingly.

Experiment 1b

A one-way ANOVA with Feedback type as a between-subject factor with six levels on participants’ mean reaction time was statistically significant [F_(5, 144)_=2.69, Root MS=34.8, *p*<.05]. Next, we tested whether, in the no-gain conditions, reaction time would be sensitive to response-effectiveness (operationalized as an immediate action-contingent effect). Critically, reaction times were shorter in the Negligible monetary no-gain (M=451, SD=31) and the Substantial monetary no-gain (only in a one-tailed test) conditions (M=458, SD=23), compared to the No-effect condition (M=475, SD=44) [*t*_47_=2.15, *p*=.03, BF_10_=1.84 (inconclusive), CI_95_ (1, 46), *d*=.63] [one tailed *t*_49_=1.68, *p*=.04, BF_10_=1.65 (inconclusive), CI_95_ (-3, 36), *d*=.48], correspondingly; and there was no significant difference between the Negligible monetary no-gain and the Substantial monetary no-gain conditions [*t*_48_=.91, *p*=.36, BF_10_=.4 (inconclusive), CI_95_ (-22, 8), *d*=.25].

Crucially, when participants could gain the money, there was clear evidence for no difference between the Substantial monetary gain condition (*M*=453, *SD*=31) and the Negligible monetary gain condition (*M*=452, *SD*=41) [*t*_47_ =.13, *p*=.89, BF_10_=.28 (conclusive), CI_95_ (-19, 22), *d* =.02]. There was no significant difference between both Monetary gain conditions (*M*=453, *SD*=36) and the Effect (*M*=440, *SD*=33) condition [*t*_73_ =.1.45, *p*=.14, BF_10_=.6 (inconclusive), CI_95_ (-29, 4), *d* =.37].

Experiment 2a

As in the former experiment, no difference in participants’ RT was found between the Substantial (M=510, SD=38) and Negligible (M=512, SD=28) monetary gain conditions [*t*_(92)_=.23, *p*=.81, BF_10_=.22 (conclusive), CI_95%_(-12, 15), *d* =.05].

Experiment 2b

No difference in participants’ RT was found between the Substantial (M=497, SD=50) and Negligible (M=498, SD=68) monetary gain conditions [*t*_(27)_=.05, *p*=.95, BF_10_=.35 (inconclusive), CI_95%_(-11, 18), *d* =.01]. In addition, RT in both Substantial [*t*_(24)_=1.27, *p*=.21, BF_10_=.8 (inconclusive), CI_95%_(-20, 86), *d* =.56] and Negligible [*t*_(27)_=1.50, *p*=.14, BF_10_=.65 (inconclusive), CI_95_ (-11, 74), *d* =.5] monetary conditions were nominally *slower* than the No-effect condition (M=465, SD=63).

**Self-reported belief of receiving the monetary reward**

In Exp.1b and 2a we also asked participants at the end of the experiments whether they believed they would receive the monetary reward they had gained at the end of the experiments having believed that this may modulate the effect of monetary reward. Unfortunately, such a question about their belief may have raised participants’ suspicion about receiving the monetary reward thus, reported-disbelief may also reflect participants’ attempt at not being perceived as naïve. Accordingly, the validity of the following analyses is low. Moreover, it should be noted that in previous experiments using this task (e.g., Karsh & Eitam, 2015a), RT was repeatedly found to be insensitive to explicit judgments, explicit knowledge and perceived control.

Twenty-four participants (~50%) from Exp. 1b and forty-seven participants (~50%) in Exp.2a, reported they did not believe they would gain the monetary compensation. In the following analyses we used participants’ self-reported belief as additional predictor to examine its contribution to the speed of response selection. Given the similar suspicion rates across both experiments and for better estimation of the parameters, the following analyses were conducted on pooled data from both experiments.

We regressed RT on both Monetary value (Substantial vs. Negligible), self-reported Belief in that the reward will indeed be given (yes or no) and their interaction while controlling for the variability explained by the individual experiments. Over the individual experiments (Exp.1b and 2a), monetary value did not predict RT [(*p*=.72, β=-.03, CI_95_(-19, 13)] nor participants’ Belief [(*p*=.75, β=-.02, CI_95_(-18, 13)]. There was also no significant contribution for their interaction to participants’ RT [(*p*=.70, β=.04, CI_95_(-18, 27)].

Further exploration of the data (which was admittedly driven by our prior naïve belief that monetary value ‘must’ have an effect on RT), revealed that after excluding both none native Hebrew speakers and participants who reported not to believe they would gain the monetary reward from the analysis, RT in Exp. 1b was only nominally shorter in the Substantial (M=434, SD=22) compared to the Negligible (M=457, SD=28) monetary gain condition [*t*_(17)_=1.97, *p*=.06, CI_95%_(-1, 48), *d* =.9]. However, this pattern, using the same filtering procedure, was not replicated in Exp. 2a; specifically, RT did not differ between the Substantial (M=509, SD=34) and the Negligible (M=508, SD=27) monetary gain conditions [*t*_(41)_=-.11, *p*=.90, CI_95%_(-20, 17), *d* =.03]. There was also no indication of a reliable contribution of monetary value on RT when the pooled data from both experiments (Exp.1b and 2a) was used while controlling the variability explained by the individual experiments [(*p*=.41, β=-.07, CI_95_(-21, 8)]. Thus, the pattern of results suggests that participants’ self-reported belief of receiving the monetary compensation has no reliable effect on their speed of responding. Hence, we did not include this item in any of the following experiments (Exp. 2b-4). However, in the following experiments we attempted to further increase participants’ ‘belief’ by verbally emphasizing the fact that they could gain additional payment (according to the assigned condition) at the end of the task.
